# Spirituality and anxiety in pastoral care workers and physicians in the first wave of the COVID-19 pandemic

**DOI:** 10.3389/fpsyt.2024.1354044

**Published:** 2024-03-11

**Authors:** Anna C. Culmann, Andreas M. Baranowski, Julia-K. Matthias, Simone C. Tüttenberg, Wilfried Belschner, Yesim Erim, Eva Morawa, Petra Beschoner, Lucia Jerg-Bretzke, Christian Albus, Susann Steudte-Schmiedgen, Martin Reuter, Franziska Geiser

**Affiliations:** ^1^ Department of Psychosomatic Medicine and Psychotherapy, University of Bonn, Medical Faculty and University Hospital, Bonn, Germany; ^2^ Department of Psychology, University of Bonn, Bonn, Germany; ^3^ Department of Psychology, Carl von Ossietzky University of Oldenburg, Oldenburg, Germany; ^4^ Department of Psychosomatic Medicine and Psychotherapy, University Hospital of Erlangen, Friedrich-Alexander University Erlangen-Nürnberg (FAU), Erlangen, Germany; ^5^ Department of Psychosomatic Medicine and Psychotherapy, Ulm University Medical Center, University Ulm, Ulm, Germany; ^6^ Department of Psychosomatic Medicine and Psychotherapy, Hospital Christophsbad, Göppingen, Germany; ^7^ Department of Psychosomatics and Psychotherapy, University of Cologne, Medical Faculty and University Hospital, Cologne, Germany; ^8^ Department of Psychotherapy and Psychosomatic Medicine, Faculty of Medicine, Dresden University of Technology, Dresden, Germany

**Keywords:** anxiety, generalized anxiety, spirituality, transpersonal trust, sense of coherence, protective factor

## Abstract

**Background:**

The COVID-19 pandemic had serious impact on the well-being of health care workers and highlighted the need for resources to help hospital staff to cope with psychologically negative consequences. The purpose of this study was to investigate the potentially protective effect of spirituality, as measured by the construct of transpersonal trust, against anxiety in physicians and in hospital pastoral care workers. In addition, transpersonal trust was compared to the effects of other potential resources, namely sense of coherence, optimism, and resilience. We also explored the relationship between transpersonal trust and anxiety and how it was moderated by sense of coherence and expected a significant effect.

**Method:**

The sample included *N* = 405 participants (*n* = 151 pastoral care workers and *n* = 254 physicians) who completed an online survey during the first wave of the COVID-19 pandemic between 20th April and 05th July, 2020, that comprised established questionnaires assessing anxiety, transpersonal trust, sense of coherence, and resilience.

**Results:**

There was no statistically significant negative relationship between transpersonal trust and anxiety in either profession or broken down by occupational group. Multiple regression analysis revealed that sense of coherence inversely predicted generalized anxiety, while transpersonal trust, resilience, and optimism did not. As hypothesized, the association between transpersonal trust and anxiety was moderated by sense of coherence. However, we could not confirm our hypothesis of a protective effect of transpersonal trust against anxiety.

**Conclusion:**

Our results point to the significant role of sense of coherence as a protective factor against anxiety and highlight the complexity of the relationship among spirituality, transpersonal trust, and anxiety.

## 1 Introduction

The COVID-19 pandemic has resulted in serious problems related to the mental well-being of health care workers (HCW) and has highlighted the need to identify resources that could help HCW mitigate psychologically adverse responses. Specifically, HCW worldwide, including those in Germany, faced devastating psychological distress such as symptoms of anxiety and depression (1–8). However, hospital pastoral care workers (PCW) received little attention in previous research on the impact of the pandemic. Additionally, some PCW were declared heroes together with health care colleagues, but others were perceived merely as infection risks (9); research showed that some PCW were valued and effectively deployed during the pandemic (10), while others reported that their employers had told them not to come to work (11).

In addition to the burdens of HCW work during COVID-19, the pandemic also brought up the question of protective factors to the center of attention. A large meta-analysis on studies conducted during the pandemic showed that perceived external resources, perception of control, positive cognitive and behavioral coping, positive outcome expectancy and meaning, coherence and spirituality promote resilience (12). In HCW, self-efficacy and optimism have been shown to be protective resources (13, 14), but few researchers explicitly studied religious and spiritual beliefs in HCW during the pandemic (15).

In general, previous research on the relationship between religiosity or spirituality and anxiety in a variety of samples revealed mixed results. In research from primarily Western countries with a predominantly Christian population, Koenig (16) discovered that 147 (49%) studies reported an inverse relationship, while 33 (11%) reported greater anxiety in individuals with high religiosity or spirituality scores. Findings from the Middle East with a predominantly Muslim population paint a similar picture (17). Ano and Vasconcelles (18) found in their meta-analysis, that people who reported using negative forms of religious coping, such as defining stressors as a punishment from God for sins, experienced increased levels of depression, anxiety, and distress.

A large proportion of studies on the effects of spirituality or religiosity on anxiety levels in HCW during the COVID-19 pandemic emphasized their positive effects on mental health. In their review, Diego-Cordero et al. (19) found that spirituality was an important coping mechanism for HCW during the pandemic, promoting mental health and well-being. At the same time, a number of authors reported negative correlations between spirituality or religiosity and anxiety during COVID-19 (20–25), while some obtained no significant relationship between religiosity and anxiety (15). Other authors also did not find a significant association for COVID-19-related fear (26) and exhaustion (27). However, further authors found that negative religious coping elevated anxiety (28, 29).

Spirituality generally encompasses individual beliefs, ways of being, and practices aimed at attaining an experience of or unity with the transcendent or divine. Religion is a phenomenon that binds people together through shared belief systems and practices in pursuit of a connection to the divine. Beliefs, practices, and rituals can incorporate both spiritual and religious aspects (30). Spirituality or religiosity can be operationalized through the concept of transpersonal trust. Transpersonal trust refers to spiritual experiences of persons who witness the existence of a higher reality, trust it, and experience a strong connection with it (31). It reflects a dimension of trust in processes of life, in a higher purpose of life, or a higher power such as God (31, 32).

Belschner (31) hypothesized that psychotherapeutic treatment and psychiatric rehabilitation would be more effective if they combined the life form of doing (i.e., self-efficacy) and the life form of letting go (i.e., transpersonal trust), but he found treatment effects only in individuals who scored high on both dimensions. Furthermore, he (32) highlighted a strong connection of transpersonal trust to the concept of salutogenesis (33), which includes the key component of sense of coherence (SOC). The coherence hypothesis by Idler (34), which states that religion may benefit health insofar as it provides SOC and meaning (35), could explain the health benefits of religiosity. George et al. (36) later extended the hypothesis to describe that Antonovsky’s SOC mediates religiosity/spirituality and mental health.

According to Antonovsky (37), SOC consists of three elements, namely comprehensibility, manageability, and meaningfulness. It focuses on the ability of people to understand the respective situation they find themselves in as well as to discover meaning and have the capacity to act in a way that may promote health (37, 38). In their systematic review, Eriksson and Lindström (39) revealed, that a higher SOC was negatively related to depression, anxiety, and post-traumatic stress. Research on HCW has also found, that a higher SOC leads to fewer mental health problems (15, 40–43).

The aim of our study was to investigate the potential protective effect of transpersonal trust against anxiety in physicians as well as in PCW working in hospitals during the COVID-19 pandemic in Germany; this crisis was existentially challenging for both professions. Transpersonal trust is a core concept in the work of the PCW, but less so for physicians; nonetheless, it could be an important protective factor against anxiety for both groups. To our knowledge, our study is the first to measure and compare transpersonal trust in both professional groups.

First, we were interested in whether transpersonal trust differs between PCW and physicians. Based on previous findings on religiosity and spirituality in HCW (44), we hypothesized that PCW would report higher levels of religiosity and spirituality than physicians. In addition, we expected to replicate previous findings on the negative association between spirituality and anxiety (19) as well as between SOC and anxiety (e.g., Schmuck et al. (15)) during the COVID-19 pandemic, and hypothesized a positive association between transpersonal trust and SOC based on Belschner’s (32) assumption of a strong connection between these two concepts. Furthermore, we were interested in how transpersonal trust relates to anxiety as opposed to other resource factors, such as SOC, resilience, and optimism. Finally, we sought to investigate whether SOC moderated the relationship between transpersonal trust and anxiety. Based on the proximity of the construct transpersonal trust to Antonovsky’s (33) SOC postulated by Belschner (32) and the sense of coherence hypothesis (36) we assumed a significant effect.

## 2 Materials and methods

### 2.1 Data collection

We utilized data from Bonn as part of the VOICE study, a large prospective multicenter cohort study involving the psychosomatic departments of the university hospitals in Bonn, Erlangen, Ulm, Cologne, and Dresden. The study seeks to uncover distress and resources in HCW during the pandemic. As the full versions of the questionnaires on transpersonal trust, SOC, and resilience were only included in the survey conducted at the university hospital in Bonn, we worked exclusively with data from Bonn. We recruited HCW and administered the survey from April 20th to July 05th, 2020, via a link we provided via intranets and internal mailing lists of the five participating university hospitals. We also contacted other hospitals and asked them to forward our study to their employees. Various medical professional associations also supported us in disseminating the study invitation (e.g., Bavarian General Practicioners’ Association, Federal Working Group of the Social Pediatric Centers, Federal Association of Psychosomatics and Medical Psychotherapy, Federal Association of Occupational Medicine and Coliquio, an internet platform for physicians). We recruited PCW via mailing lists from the national chair of Catholic hospital chaplaincy and the German Society for Pastoral Psychology. The Ethics Committee of the Medical Faculty of the University of Bonn (reference number: 125_20) approved the study, and all respondents provided informed consent online.

The survey was administered via the academic survey tool SoSci Survey (www.soscisurvey.com). The survey comprised 82 items and took approximately 20 minutes to complete. Inclusion criteria were a minimum age of 18 years, working in a German hospital, medical care center or in a private practice, having a residence/working place in Germany, and sufficient knowledge of German language. In the analyses reported here, we only included participants who worked as a physician or a PCW, and provided complete data sets. PCW in Germany are clergy or spiritual care workers who are sent or seconded to hospital work by religious communities or churches. The two dominating churches in Germany are the Roman Catholic church (comprising 25% of the German population) and the Protestant church (23% of population); 44% of the German population declare not to be member of a religious community (45). PCW are paid by their churches but are organizationally integrated into the hospital. The survey was anonymous.

### 2.2 Sample characteristics

A total of *N* = 1,232 HCW participated in our online survey. In this study, we only analyzed data from PCW and physicians. Of the *N* = 427 respondents (*n* = 272 physicians and *n* = 155 PCW), 22 respondents had to be excluded from subsequent analyses because of incomplete data sets. Thus, the final sample size was a total of *N* = 405 participants (*n* = 254 physicians and *n* = 151 PCW). The online survey included sociodemographic variables (gender, age category, living alone, caregiving responsibilities to relatives, children, single parent, migration background), occupational variables (work setting, profession, years of professional experience, working hours), and a range of COVID-19 related variables. For our analyses, we focused on age group, gender, professional experience, and profession. A detailed overview of age, gender, and professional experience distribution of the two occupational groups is given in **Table 1**.

**Table 1:** Descriptive statistics for sociodemographic and occupational variables for physicians and PCW.

	Physicians (*n* = 254)	PCW (*n* = 151)	Total sample (*N* = 405)
Gender, *n* (%)			
Male	93 (36.6)	63 (41.7)	156 (38.5)
Female	160 (63.0)	88 (58.3)	248 (61.2)
Diverse	1 (0.4)		1 (0.2)
Age, years, *n* (%)			
18-30	50 (19.7)	0 (0)	50 (12.3)
31-40	80 (31.5)	3 (2.0)	83 (20.5)
41-50	57 (22.4)	17 (11.3)	74 (18.3)
51-60	49 (19.3)	95 (62.9)	144 (35.6)
61-70	18 (7.1)	34 (22.5)	52 (12.8)
>70	0 (0)	2 (1.3)	2 (0.5)
Professional experience, *n* (%)			
<3 years	47 (18.5)	22 (14.6)	69 (17.0)
3-6 years	33 (13.0)	19 (12.6)	52 (12.8)
>6 years	169 (66.5)	81 (53.6)	250 (61.7)
unknown	5 (2.0)	29 (19.2)	34 (8.4)

### 2.3 Measures

#### 2.3.1 Anxiety

We measured anxiety with the Generalized Anxiety Disorder Scale-2 (GAD-2) (46) that includes two items; one question is “Over the last two weeks, how often have you been bothered by the following problems? - Feeling nervous, anxious or on edge.” The four-point Likert scale ranges from 0 (“not at all”) to 3 (“nearly every day”). The GAD-2 is internationally validated (47), and the psychometric criteria of the scale have been well studied (46). In our survey, it achieved an acceptable Cronbach’s α of.73.

#### 2.3.2 Transpersonal trust

We used the Transpersonal Trust Questionnaire (TPV) (48) to assess religiosity and spirituality. The scale describes a person who recognizes the existence of a higher reality, trusts it, and experiences a strong connection with it (31). It was measured on a representative sample of the German population (49) and used to predict treatment success in the psychotherapeutic setting (31). An exemplary item is “I feel connected with a higher reality/with a higher being/with God. Even in hard times I can trust this reality.” The scale consists of eleven items, which are rated on a four-point Likert scale ranging from 0 (“does not apply at all”) to 3 (“applies completely”). With a calculated reliability of Cronbach’s α = .92 in our sample, the questionnaire has excellent internal consistency.

#### 2.3.3 Sense of coherence (SOC)

Antonovsky’s (33) concept of SOC is composed of the three elements comprehensibility, manageability, and meaningfulness and formed the basis of the SOC-13 (50), which we used for this study. The SOC-13 is an economical instrument with good reliability (51) with five items being inverted. The items were rated on a seven-point Likert scale ranging from 1 (“very often”) to 7 (“very seldom or never”). One example item is “Do you have very mixed-up feelings and ideas?” Internal consistency in the present sample was good with Cronbach’s α = .83.

#### 2.3.4 Resilience

Psychological resilience was assessed by the five-item Resilience Scale (RS-5) (52), which is the short version of the original 25-item Resilience Scale (RS) (53). It is important to note that the concept of resilience is inherently heterogeneous (54). The authors of the questionnaire define resilience as “a positive personality characteristic that enhances individual adaption”, which consists of two dimensions, acceptance of self and life and personal competence (53). The RS-5 was validated based on excellent goodness-of-fit criteria (55); a sample item is “Keeping interested in things is important to me.” Responses can be given on a seven-point Likert scale from 1 (“No, I disagree”) to 7 (“Yes, I completely agree”). In our study, Cronbach’s α was .78, which represents sufficient reliability.

#### 2.3.5 Optimism

Following Kemper et al. (56), we measured optimism with the item “How optimistic are you in general?”, which can be answered on a seven-point Likert-scale from 1 (“not optimistic at all”) to 7 (“very optimistic”). Higher values reflect higher levels of optimism.

### 2.4 Statistical analyses

Data analyses were performed using IBM SPSS Statistics (Version 28) and R (Version 4.1.1) with significance set at *p* <. 05. To account for multiple testing, the *p*-value for correlation analyses was adjusted to .017, using Bonferroni correction. To test for equal frequency distribution in gender, a Pearson χ²-test was calculated. The MANCOVA was conducted to compare means between groups. Furthermore, Pearson correlations were conducted to analyze relationships between variables. To explore further multivariate relationships between variables, we performed multiple regression (inclusion method) and a moderation analysis. The moderation analysis was conducted using Hayes’ (57) PROCESS macro, which uses ordinary least squares regression, yielding unstandardized coefficients for all effects. We used bootstrapping with 5000 samples together with heteroscedasticity-conforming standard errors (58) to calculate confidence intervals. As effect size measures, we report Cohen’s *d* for *t*-tests and partial η^2^ for multivariate analyses of covariance (MANCOVA).

## 3 Results

### 3.1 Characteristics of the sample

Descriptive statistics for sociodemographic and occupational variables for physicians and PCW are presented in **Table 1**. Sex was equally distributed across physicians and PCW with about 40% male and 60% female participants. The average age of PCW was significantly higher than that of physicians, *t*(403) = −13.80, *p* <. 001, *d* = 1.04. With 62.9%, considerably more than half of the PCW were between 51 and 60, while 31.5% of physicians were between 31 and 40, and only 19.3% were between 51 and 60 years old. Across both occupational groups, 61.7% of the respondents had more than six years of work experience, and 17.0% had less than three years of work experience. Another 19.2% of PCW stated that they were not involved in direct patient care.

### 3.2 Correlations between generalized anxiety, transpersonal trust, and SOC

Correlation analyses revealed that generalized anxiety and transpersonal trust were not significantly negatively correlated across the total sample (*r* = −.03, *p* = .279). Within the respective professional groups, the correlations were *r* = .02, *p* = .366 for physicians and *r* = .04, *p* = .300 for PCW. In contrast, generalized anxiety and SOC were significantly negatively correlated across both groups (*r* = −.52, *p* <. 001); here, the correlation for physicians was *r* = −.55, *p* <.001 and *r* = −.41, *p* <.001 for PCW. Transpersonal trust and SOC were significantly positively correlated only in the total sample (*r* = .14, *p* = .003), but not in the respective occupational groups. The correlations were *r* = .04, *p* = .276 for physicians and *r* = .12, *p* = .070 for PCW.

### 3.3 MANCOVA of generalized anxiety, transpersonal trust, SOC, optimism, and resilience between occupational groups

To compare transpersonal trust, SOC, optimism, resilience, and generalized anxiety between physicians and PCW, we calculated a one-factor MANCOVA. Because the two groups differed significantly in age, caregiving responsibilities to relatives, contact with infected individuals, and working hours, we used these four variables as covariates. The MANCOVA showed a statistically significant difference between PCW and physicians for the combined dependent variables, *F*(5, 395) = 28.65, *p* < .001, η_p_^2^ = .27, Wilks Λ = .73.

There was a statistically significant difference between PCW (*M* = 3.30, *SD* = 0.41) and physicians (*M* = 2.25, *SD* = 0.74) for transpersonal trust (*F*(1, 399) = 131.48, *p* < .001, η_p_^2^ = .25). Scores for generalized anxiety (*F*(1, 399) = .01, *p* = .916, η_p_^2^ <.01), SOC (*F*(1, 399) = .64, *p* = .423, η_p_^2^ <.01), optimism (*F*(1, 399) = 2.05, *p* = .153, η_p_^2^ = .01), and resilience (*F*(1, 399) = 2.24, *p* = .135, η_p_^2^ = .01) did not differ significantly between groups. All means and standard deviations are presented in **Table 2**.

**Table 2:** Means and standard deviations for transpersonal trust, SOC, optimism, resilience, and generalized anxiety, based on the MANCOVA.

	Physicians (*n* = 254)	PCW (*n* = 151)	Total sample (*N* = 405)
Transpersonal trust (TPV), mean (*SD*)	**2.25 (0.74)**	**3.30 (0.41)**	2.64 (0.82)
Sense of coherence (SOC-13), mean (*SD*)	5.10 (0.87)	5.35 (0.73)	5.19 (0.83)
Optimism, mean (*SD*)	5.09 (1.39)	5.36 (1.26)	5.19 (1.34)
Resilience (RS-5), mean (*SD*)	5.84 (0.86)	5.84 (0.77)	5.84 (0.83)
Generalized anxiety (GAD-2), mean (*SD*)	1.68 (0.70)	1.57 (0.54)	1.64 (0.65)

Significant group differences are printed in bold.

### 3.4 Multiple linear regression of generalized anxiety

In a next step, we conducted a multiple linear regression to measure the extent to which the predictors transpersonal trust, SOC, optimism, and resilience explained variance in the criterion generalized anxiety. The model showed a substantial amount of explained variance with *R*² = .28 (corrected *R*² = .27). When combined, the four variables significantly predicted anxiety (*F*(4, 400) = 38.22, *p* < .001), although individually, only SOC significantly predicted anxiety. An overview of the coefficients and significance of predictors can be found in **Table 3**.

**Table 3:** Coefficients and significance of predictors of multiple linear regression.

	Regression coefficient (*b*)	Standard error (*SE*)	Beta (β)	*T*	*p*
Transpersonal trust	.03	0.03	.04	0.94	.349
Sense of coherence	-.41	0.04	-.53	-10.22	< .001
Optimism	.03	0.02	.06	1.24	.215
Resilience	-.05	0.04	-.07	-1.36	.174

Criterion variable = generalized anxiety.

Separate analyses conducted for each occupational groups revealed that the regression model was significant for both PCW (*R*² = .19, corrected *R*² = .17) and physicians (*R*² = .33, corrected *R*² = .32). SOC was a more influential predictor in physicians (β = −.57) than in PCW (β = −.38), and was again in both groups the only significant predictor of anxiety; for PCW with *T* = −4.35, *p* < .001, for physicians with *T* = −8.92, *p* < .001).

### 3.5 Moderation analysis of SOC on the association between transpersonal trust and generalized anxiety

We assumed that the effect of transpersonal trust on anxiety was conditional on SOC and therefore conducted a moderation analysis. First, we calculated the interaction effect, which represents the moderating influence of SOC on the relationship between transpersonal trust and generalized anxiety. The overall model was significant, *F*(4, 400) = 29.23, *p* < .001, with a high amount of explained variance 29.24%. The results showed that SOC significantly moderated the effect between transpersonal trust and generalized anxiety, Δ*R*² = 1.70%, *F*(1, 400) = 6.40, *p* = .012, 95% CI [0.03, 0.24].

**Figure 1:**
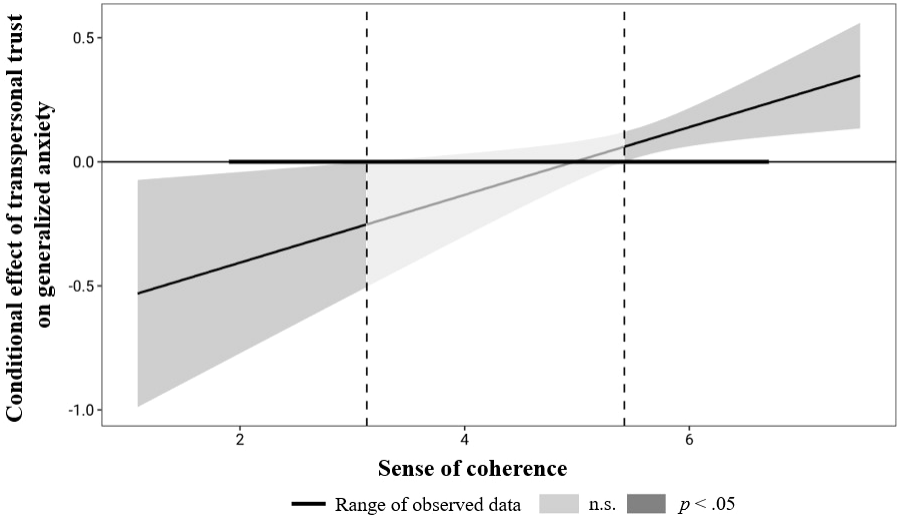
Johnson-Neyman plot of the interaction effect of transpersonal trust and SOC on generalized anxiety across both professional groups of physicians and PCW.

Next, we computed a Johnson–Neyman plot (**Figure 1**), which allowed us to identify the specific regions of significance for the moderator variable: If the moderator SOC is outside the interval [3.13, 5.42], the conditional effect of transpersonal trust on anxiety is significant (*p* < .05). Unexpectedly, very high SOC had a significantly positive effect on the relationship between transpersonal trust and anxiety. In accordance with our hypothesis, low SOC had a significantly negative effect on the relationship.

## 4 Discussion

The aim of our study was to investigate the potentially protective effect of transpersonal trust against anxiety in physicians and PCW during the first wave of the COVID-19 pandemic. First, we found support for our hypothesis that transpersonal trust would be higher among PCW than among physicians, which supported earlier findings of higher levels of spirituality and religiosity among PCW and lower levels among physicians (44). Anxiety, SOC, optimism, and resilience did not differ significantly between the two groups.

We could not confirm our hypothesis of a significant negative correlation between spirituality, as measured by transpersonal trust, and anxiety. This finding was initially surprising, as a majority of research on the relationship between spirituality or religiosity and anxiety in times of COVID-19 pandemic supported the protective character of spirituality or religiosity. However, others found no significant correlation between religiosity and anxiety (15, 26) or even found that negative religious coping elevates anxiety (28, 29). Koenig (16) also highlights the inconsistency and heterogeneity of findings on the relationship. One possible explanation for the nonsignificant correlation between transpersonal trust and anxiety could be the specialization of religiosity and spirituality scales (59). The TPV operationalizes primarily the intensity of spirituality (59) in context of a connection with a higher power (31) as a specific aspect of spirituality that could be independent from anxiety. Furthermore, the result is consistent with the finding, that spirituality as an attitude or belief, as described by the TPV, only had a small protective effect, whereas spiritual experiences may be beneficial (60).

Our multiple regression analysis revealed that SOC explained the largest amount of variance in generalized anxiety, whereas transpersonal trust, optimism, and resilience explained only little variance. In a very large German sample recruited during the COVID-19 pandemic that were also our data source, SOC, as measured by a very short three-item scale (61), was also associated with lower anxiety (15). Meanwhile, other researchers found associations between high SOC and fewer mental health problems in HCW (40, 42, 43, 62) as well as in the general population (39).

The moderation analysis provided surprising results: Low SOC moderated a significant negative correlation between transpersonal trust and anxiety; the effect was negative but nonsignificant with moderate SOC; and very high SOC was associated with a significant positive correlation between transpersonal trust and anxiety. The negative correlation between the two variables with low SOC could be explained with Belschner’s (32) emphasis on the relationship between the two constructs of transpersonal trust and SOC; transpersonal trust can temper high anxiety in individuals who perceive challenges as less comprehensible, manageable, or meaningful. Another possible explanation is that people who experience little control themselves, and thus have comparatively low SOC, can easily entrust control to a higher being. Following this line of argument, it seems reasonable that with low SOC, transpersonal trust is associated with less anxiety.

However, the significant positive correlation between transpersonal trust and anxiety with high SOC, is more difficult to explain. A possible, though somewhat speculative explanation, is that in persons with high SOC and high transpersonal trust, the crisis was more devastating to their faith, and created inner conflict and thereby anxiety. Interestingly, researchers found that people associated COVID-19 infection with doubts regarding the power of God (63). A further cautious interpretation of this result is that if the situation is already perceived as comprehensible, manageable and meaningful, the ability to trust nevertheless leads to allowing more fear in addition to active coping, since anxiety is secured in a higher instance.

Our study provides exciting results on the relationships of spirituality/religiosity and SOC with anxiety including the moderating effect of SOC. We were the first to use such a large sample of PCW in the context of the COVID-19 pandemic to examine this relationship. The results suggest that transpersonal trust alone cannot initially be considered a protective factor against anxiety. Our results support Belschner’s (31) finding that transpersonal trust as a sole factor does not lead to a better treatment effect. However, Belschner also found that high expressions of self-efficacy (64) and transpersonal trust (32) improved treatment outcome (31), yet we did not find such correlations. Future researchers may examine the relationship between transpersonal trust and other concepts of coping such as self-efficacy to gain a more detailed understanding.

One limitation of our study is the cross-sectional design, which does not allow for conclusions about the interrelationships between generalized anxiety, transpersonal trust, SOC, optimism, and resilience. Some of our propositions for the explanation of the moderation effect we obtained imply a causal effect of SOC or transpersonal trust on anxiety, which may be supported by earlier research, but remains speculative given that it is based on simple correlations. A second limitation is the use of very short scales to measure generalized anxiety (two items) and optimism (one item), which we chose due to the necessity for brevity to suit healthcare professionals’ time constraints. Furthermore, the use of the screening instrument GAD-2 with its two items inevitably leads to an overestimation of anxiety in our sample, albeit a moderate one. There might also be a nationality effect. In particular, findings on spirituality and religiosity and its relation to anxiety have been primarily studied in the United States (see e.g. the review article by Koenig (16)). It is possible and likely that religiosity and spirituality have different forms and effects in a largely secular European society compared to more religiously bound American contexts (65, 66), which limits international comparisons.

In summary, our hypothesis that transpersonal trust alone could be a protective factor against anxiety in physicians and PCW during the COVID-19 pandemic was not confirmed. Rather, the results point to the significant role of SOC as a protective factor against anxiety and highlight the complexity of the relationship between transpersonal trust and anxiety. The findings indicate that individuals who exhibit low levels of anxiety tend to possess either elevated transpersonal trust or a strong SOC.

One implication for future studies is the extensive testing of the sense of coherence hypothesis (34, 36). For example, further constructs of mental health could be included in the calculations, such as depression or PTSD, longer questionnaires could be applied and both moderation and mediation analyses could be calculated. Furthermore, it would be possible to explore the extent to which the results are the same or different outside the pandemic context and whether the pattern of results can also be found in other occupational groups in the healthcare system. An inclusion of other religiosity and spirituality scales with a different focus than the TPV could also provide interesting results. The finding that, in our sample, optimism and resilience explained very little variance in anxiety, also remains an open question for future researchers to answer.

Our study’s findings reveal practical implications, with SOC emerging as a significant protective factor against anxiety, emphasizing the importance of developing tailored trainings and workshops to enhance SOC among HCW. The findings also suggest the potential benefit of integrating spiritual support with psychological interventions, especially considering the unique stresses faced by PCW and physicians during pandemics. This approach highlights the necessity for healthcare institutions to provide targeted mental health support and develop brief, validated instruments for rapid assessment in crisis situations. By promoting a variety of positive coping mechanisms tailored to diverse beliefs and preferences, healthcare settings can better support their staff in managing stress and anxiety effectively.

## Data availability statement

The raw data supporting the conclusions of this article will be made available by the authors, without undue reservation.

## Ethics statement

The studies involving humans were approved by Ethics Committee of the Medical Faculty of the University of Bonn. The studies were conducted in accordance with the local legislation and institutional requirements. The participants provided their written informed consent to participate in this study.

## Author contributions

AC: Conceptualization, Formal analysis, Methodology, Writing – original draft, Writing – review & editing. AB: Conceptualization, Methodology, Project administration, Supervision, Writing – review & editing, Formal analysis, Validation. J-KM: Writing – review & editing. ST: Validation, Writing – review & editing. WB: Writing – review & editing. YE: Writing – review & editing. EM: Writing – review & editing. PB: Writing – review & editing. LJ-B: Writing – review & editing. CA: Writing – review & editing. SS-S: Writing – review & editing. MR: Writing – review & editing, Methodology. FG: Conceptualization, Funding acquisition, Investigation, Methodology, Project administration, Supervision, Writing – review & editing.
